# Morphological and Rheological Analysis of an Injectable Calcium Hydroxylapatite Dermal Filler with Lattice-Pore Surface Architecture

**DOI:** 10.3390/jfb17090476

**Published:** 2026-09-19

**Authors:** Gi-Woong Hong, Yerin Park, Doo Yeoul Chang, Jeesoo Kook, Young Bin Lim, Ho Lee, Kyu-Ho Yi

**Affiliations:** 1 Samskin Plastic Surgery Clinic, Seoul 06577, Republic of Korea; cosmetic21@hanmail.net; 2 Medical Research Inc., Wonju 26433, Republic of Korea; 3 Change Clinic, Seoul 06054, Republic of Korea; 4 Itsme Clinic, Ansan 15360, Republic of Korea; 5 The Walts Clinic, Seoul 06134, Republic of Korea; 6 LeSommet Clinic, Seoul 06070, Republic of Korea; 7 You and I Clinic, Seoul 07996, Republic of Korea

**Keywords:** calcium hydroxylapatite, lattice pore architecture, microsphere morphology, rheology, dermal filler, biomaterials

## Abstract

**Background:** Hydroxylapatite is a biocompatible calcium-phosphate ceramic used in regenerative biomaterials. Calcium hydroxylapatite (CaHA) fillers combine immediate mechanical support with subsequent extracellular matrix remodeling, and their material behavior depends on microsphere morphology, mineral composition, carrier interactions, and rheology. **Objectives:** To determine whether a CaHA formulation prepared using Lattice Pore Formation technology (Facetem) exhibits a distinct and internally consistent material profile across microsphere geometry, lattice-pore surface architecture, mineral composition, formulation-level sedimentation, and rheological behavior across dilution ratios, with selected morphology and sedimentation characteristics compared with Radiesse. **Methods:** Facetem (marketed in the Republic of Korea as DCLASSY) was evaluated as the test product, with Radiesse used as the comparator product. Microspheres were examined using field-emission scanning electron microscopy and laser-diffraction particle-size analysis. Surface morphology was assessed before and after 12 weeks of phosphate-buffered saline (PBS) incubation. Mineral composition was evaluated using X-ray diffraction and inductively coupled plasma optical emission spectroscopy. Extrusion continuity and sedimentation after saline dilution were evaluated, and rheological properties were measured across eight dilution ratios using saline, non-cross-linked hyaluronic acid, and semi-cross-linked hyaluronic acid. **Results:** Facetem had a mean particle diameter of 34.60 μm, with higher circularity (0.95 versus 0.88) and roundness (0.96 versus 0.85), a lower aspect ratio, and fewer particles below 20 μm (2.10% versus 14.50%) than Radiesse. Micrograin domains formed a lattice-pore surface that showed morphological changes after 12 weeks of PBS incubation while the spherical contour remained recognizable. Hydroxylapatite represented 99.09% of the mineral phase, with a Ca/P ratio of 1.67. Facetem extruded as a continuous strand and showed greater supernatant clarification after dilution. Storage modulus declined with dilution, and semi-cross-linked hyaluronic acid retained more elastic resistance than saline at equivalent ratios. **Conclusions:** Facetem demonstrated a consistent microsphere population, an organized lattice-pore surface, hydroxylapatite stoichiometry, and diluent-dependent rheology. The integrated analysis defines its physicochemical profile; the PBS findings should be interpreted as morphological stability under non-biological buffer conditions rather than in vivo degradation.

## 1. Introduction

Injectable dermal fillers are widely used for soft-tissue augmentation, facial contour correction, and treatment of age-related changes in the skin and subcutaneous tissue. Hydroxylapatite is a calcium-phosphate ceramic related to the principal inorganic mineral phase of bone. Its biocompatibility, mineral stability, and capacity to provide a particulate interface have supported its use in tissue engineering and regenerative biomaterials. Recent studies have further shown that the physicochemical behavior of hydroxylapatite-based biomaterials is influenced by mineral composition, phase constitution, and microstructural characteristics [1,2]. In aesthetic formulations, calcium hydroxylapatite (CaHA) microspheres are suspended in a biodegradable gel carrier that facilitates injection and provides an initial filling effect. After the carrier is gradually absorbed, the remaining microspheres interact with surrounding cells and support fibroblast activation and formation of type I and type III collagen [3,4,5]. These characteristics have expanded the use of CaHA from localized volume restoration to structural contouring and skin-quality improvement.

The indications and expected performance of a CaHA formulation can vary according to its material characteristics. The preparation process, carrier composition, microsphere diameter, particle-size distribution, circularity, surface structure, mineral organization, and rheological properties can influence injection behavior, tissue spread, mechanical support, cellular interaction, and structural change during incubation. These parameters can also affect the anatomical layer in which the material is placed, the degree of projection or diffusion achieved after injection, and its suitability for structural contouring or more diffuse skin treatment. Previous comparative studies have reported differences among CaHA formulations in microsphere morphology, surface regularity, rheological behavior, handling characteristics, and in vitro biostimulatory activity [6,7].

Microsphere morphology is particularly important because mean particle diameter alone does not fully describe the structural properties of a particulate filler. Distribution width, circularity, roundness, aspect ratio, the proportion of small particles, specific surface area, and surface texture provide additional information on particle uniformity and behavior. Irregular contours, fractures, and broad size distributions can affect dispersion within the carrier and interaction with surrounding cells. In contrast, consistently spherical microspheres can form a more uniform particulate structure. The arrangement of the mineral phase at the surface can also influence how microspheres change during controlled incubation and, in biological settings, during resorption. A comprehensive evaluation should therefore include both quantitative particle measurements and high-magnification analysis of surface architecture [8,9].

The behavior of the complete formulation also depends on interactions between the microspheres and the gel carrier. Particle size, density, surface characteristics, particle concentration, and carrier viscosity can influence dispersion during storage, extrusion, and dilution. Sedimentation after dilution can provide information on formulation-level separation under standardized conditions, but it is affected by several variables simultaneously and should not be treated as a direct proxy for in vivo stability or as evidence that a single physicochemical factor is responsible.

Dilution further changes the mechanical and clinical profile of CaHA. Undiluted and minimally diluted formulations generally retain greater elastic resistance and are commonly used when localized support and contour definition are required. As dilution increases, the concentration of microspheres per unit volume, storage modulus, and viscosity decrease, allowing the material to spread over a broader tissue area [10,11]. The resulting mechanical properties are determined by both the dilution ratio and the characteristics of the added material. Previous rheological studies have shown that even small volumes of an aqueous diluent can substantially reduce storage modulus, loss modulus, viscosity, and cohesivity [12,13]. At the same dilution ratio, saline and a viscoelastic hyaluronic acid formulation can therefore produce markedly different mechanical profiles and potentially support different treatment objectives.

Previous comparative studies have documented substantial differences among CaHA dermal fillers in microsphere morphology, surface regularity, fragmentation, and particle size distribution, despite their shared CaHA composition [6,7,9]. Rheological properties also vary among formulations, and changes in dilution or carrier composition can markedly alter storage modulus and viscosity [12,13]. Together, these findings indicate that the physicochemical properties of a CaHA filler depend on both formulation characteristics and the manufacturing process and cannot be fully defined by CaHA content or mean particle size alone. Accordingly, a CaHA formulation produced using a distinct Lattice Pore Formation process requires direct characterization of its microsphere geometry, surface architecture, mineral composition, morphological stability in PBS, suspension and extrusion behavior, and dilutional rheology.

Demand for injectable biostimulatory materials is increasing globally. The 2024 International Society of Aesthetic Plastic Surgery survey estimated 418,173 CaHA procedures performed worldwide by plastic surgeons, representing a 13.7% increase from 2023 and an 87.7% increase from 2020 [14]. This growth increases the need for transparent, formulation-specific physicochemical characterization.

The formulation evaluated in this study, Facetem (marketed in the Republic of Korea under the brand name DCLASSY), consists of 30% CaHA microspheres suspended in a 70% sodium carboxymethylcellulose(CMC) gel carrier. The microspheres are prepared using a proprietary Lattice Pore Formation process designed to create spherical particles with a layered micrograin surface structure. This manufacturing approach was developed to position Facetem as a next-generation CaHA formulation, with tighter control over microsphere geometry and a characteristic lattice-pore surface architecture. The primary research question was whether this preparation process yields a CaHA microsphere population with a distinct and internally consistent physicochemical profile across particle geometry, surface architecture, mineral composition, formulation-level suspension behavior, and dilutional rheology. The novelty of the study is the integrated evaluation of these domains within a single analytical framework rather than inference from isolated measurements. Selected morphological and sedimentation characteristics were compared with Radiesse, whereas the PBS-incubation and dilutional-rheology analyses were intended to characterize Facetem rather than establish head-to-head superiority.

## 2. Materials and Methods

### 2.1. Study Design

This in vitro study characterized a CaHA formulation prepared using Lattice Pore Formation technology. The analyses were organized into four complementary components.

First, Facetem was directly compared with Radiesse (Merz Aesthetics, Frankfurt, Germany) for microsphere morphology, quantitative shape measurements, particle characteristics, gross appearance after extrusion, and sedimentation behavior. Both CaHA formulations consisted of mineral microspheres suspended in a gel carrier.

Second, previously generated internal analytical data from one poly L lactic acid formulation, one polylactic acid formulation, and one polycaprolactone formulation were included as secondary material references. These data were used only to compare mean particle size, distribution span, specific surface area, and uniformity index across a broader range of particulate biostimulators.

Third, the lattice-pore surface structure, morphological changes during PBS incubation, crystalline mineral phase, elemental composition, purity, and Ca/P molar ratio of Facetem were examined separately.

Fourth, the rheological properties of Facetem were evaluated after mixing with saline, non-cross-linked hyaluronic acid, or semi-cross-linked hyaluronic acid at eight dilution ratios.

Facetem (CGBIO Co., Ltd., Seongnam, Republic of Korea) contained 30% CaHA microspheres suspended in a 70% sodium CMC gel carrier and was supplied in prefilled 0.8 mL and 1.5 mL syringes. Three independent production lots of each evaluated formulation were assessed, with three syringes obtained from each lot. No human participants, human tissues, or experimental animals were included.

### 2.2. Morphological Analysis

CaHA microspheres were separated from the carrier gel by dispersing 1.0 mL of each CaHA formulation in 40 mL of distilled water. The suspensions were centrifuged at 3000 rpm for 10 min, and the supernatant was removed. This washing procedure was repeated three times to eliminate residual carrier material. The recovered particles were then lyophilized for 24 h.

**Figure 1:**
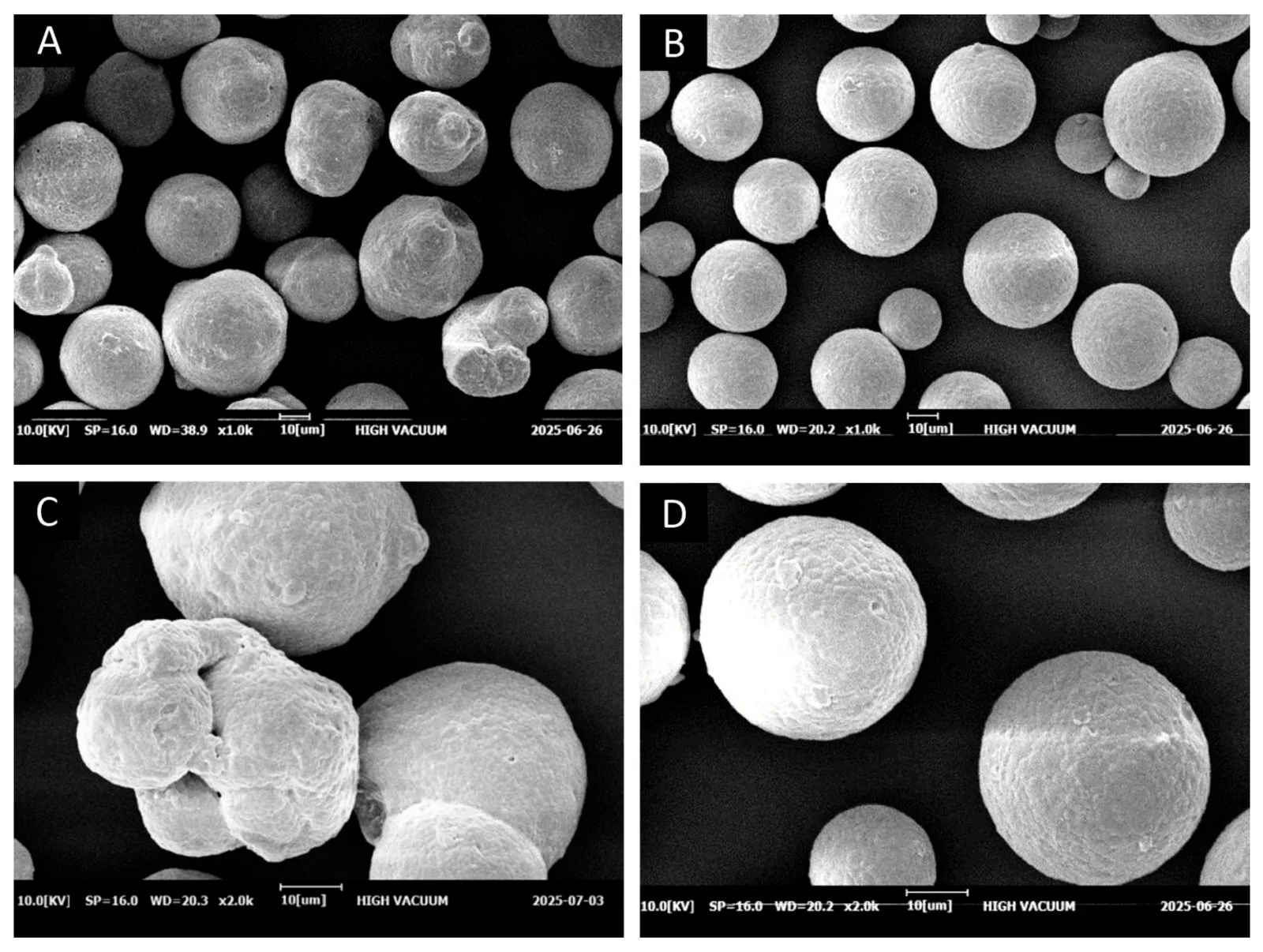
Comparative microsphere morphology of Facetem and Radiesse. Field-emission scanning electron microscopy images of CaHA microspheres after removal of the gel carrier. (**A**) Radiesse at ×1000 magnification. (**B**) Facetem at ×1000 magnification. (**C**) Radiesse at ×2000 magnification. (**D**) Facetem at ×2000 magnification. The ×5000 lattice-pore image is presented separately in Figure 2A.

**Figure 2:**
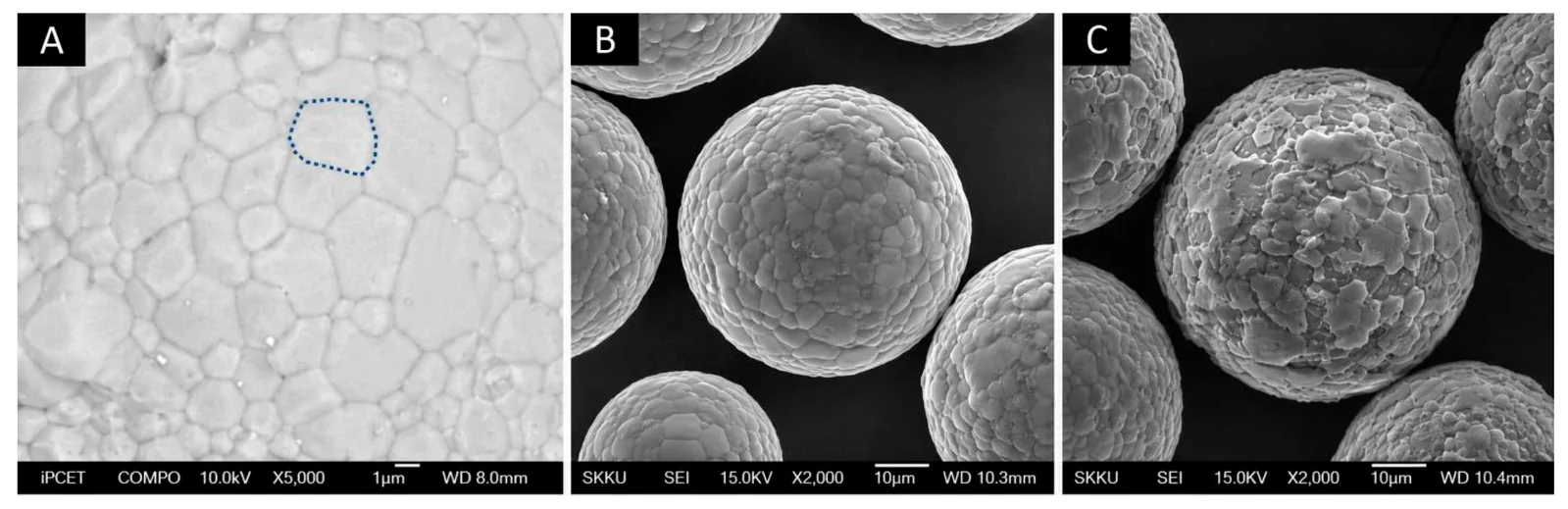
Lattice-pore surface architecture and morphological changes during PBS stability testing of Facetem microspheres. Field-emission scanning electron microscopy images of Facetem microspheres. (**A**) Lattice-pore surface architecture at ×5000 magnification. The dashed outline highlights the overlapping, layered arrangement of micrograins forming the lattice-pore surface structure. (**B**) Microspheres before PBS incubation at ×2000 magnification. (**C**) Microspheres after 12 weeks of incubation in phosphate-buffered saline at ×2000 magnification.

The dried particles were mounted on conductive stubs, coated with platinum and palladium, and examined using field-emission scanning electron microscopy (JSM-7600F, JEOL, Tokyo, Japan). Images of Facetem and Radiesse were acquired at ×1000, ×2000, and ×5000 under identical acquisition conditions. Comparative ×1000 and ×2000 images are presented in Figure 1; the ×5000 image used to characterize Facetem’s lattice-pore surface architecture is presented in Figure 2A.

Image analysis was performed using ImageJ software (version 1.54g; National Institutes of Health, Bethesda, MD, USA). At least 300 particles from each lot were assessed. Equivalent circular diameter, Feret diameter, circularity, roundness, and aspect ratio were recorded.

Circularity was calculated as 4πA/P^2^

where A represents particle area and P represents perimeter. Values approaching 1 indicate a more circular profile.

Roundness was calculated as 4A/(πFmax^2^)

where Fmax represents the maximum Feret diameter. Aspect ratio was defined as the ratio of the major axis to the minor axis. The proportion of particles with a major axis diameter below 20 μm was also calculated.

### 2.3. Particle Size and Properties

Particle size was measured using a laser diffraction particle size analyzer (Mastersizer 3000, Malvern Panalytical, Malvern, UK). Mean particle diameter, particle size range, distribution span, specific surface area, and uniformity index were recorded.

Distribution span was calculated as (D90 − D10)/D50

Specific surface area was measured using the Brunauer–Emmett–Teller method with a TriStar II surface area analyzer (Micromeritics, Norcross, GA, USA) and expressed as m^2^/kg.

The uniformity index was calculated by dividing the mean absolute deviation from the median diameter by the median diameter. Lower values indicated a narrower particle distribution around the median. Particle size histograms were prepared using 5 μm intervals. Quantitative shape measurements were compared between the two CaHA formulations. Mean particle size, span, specific surface area, and uniformity index were also examined across the CaHA, PLLA, PLA, and PCL formulations.

### 2.4. Lattice-Pore Structure and Morphological Stability in PBS

The surface of the test microspheres was examined at ×5000 to characterize the arrangement of the layered micrograin domains. Representative microspheres from Facetem were evaluated before incubation and after 12 weeks in phosphate-buffered saline at 37 °C and pH 7.4 under continuous agitation at 100 rpm. The particles were subsequently recovered, washed, dried, and examined using the same field-emission scanning electron microscopy procedure. This assay was defined as a non-biological PBS morphological-stability test; it did not include enzymes or cells and was not intended to model in vivo biodegradation. Only Facetem was assessed in this assay, and no comparative conclusion with Radiesse was drawn.

### 2.5. Mineral Composition and Purity

The crystalline mineral phase and phase purity of the test microspheres were evaluated using X-ray diffraction (SmartLab, Rigaku Corporation, Tokyo, Japan), while elemental composition and Ca/P stoichiometry were assessed using inductively coupled plasma optical emission spectroscopy (5110 ICP-OES, Agilent Technologies, Santa Clara, CA, USA). Purity was defined as the proportion of crystalline CaHA relative to the total mineral content. Calcium, phosphorus, iron, manganese, and the Ca/P molar ratio were evaluated. Macroscopic photographs of the extruded Facetem were acquired under fixed lighting, camera settings, background, and sample thickness.

### 2.6. Extrusion and Gross Homogeneity

Equal volumes of Facetem and Radiesse were extruded through a 27G needle under standardized pressure. The materials were deposited on identical nonabsorbent surfaces and photographed immediately after extrusion. Gross homogeneity was evaluated qualitatively according to the continuity of the extruded strand and visible separation between the microsphere phase and the gel carrier.

### 2.7. Sedimentation Analysis

For the primary quantitative sedimentation analysis, Facetem and Radiesse were mixed with 0.9% sodium chloride at a CaHA to saline ratio of 1:10. An additional 1:2 condition was prepared using 1.5 mL of each CaHA formulation and 3.0 mL of saline to determine whether the direction of sedimentation observed at 1:10 was maintained at a lower dilution. Each mixture was homogenized using 10 manual inversion cycles and immediately transferred into an identical transparent tube. Images were acquired at 4, 8, and 15 min under standardized lighting and camera settings. Supernatant transparency was measured by optical densitometry using a standardized region of interest in ImageJ and was expressed in arbitrary grayscale units. The percentage change from baseline was calculated as:

Transparency change (%) = [(*T*_15_ − *T*_0_)/*T*_0_] × 100

where *T*_0_ is baseline supernatant transparency and *T*_15_ is supernatant transparency at 15 min. A relative residual turbidity index was calculated by normalizing Radiesse value to Facetem value at 15 min. The 1:2 condition was assessed visually and was not included in the optical densitometry analysis. Sedimentation was interpreted as a descriptive formulation-level assay. Because paired viscosity or density measurements were not obtained for both CaHA formulations, the analysis was not used to assign causality or derive a viscosity/density–sedimentation correlation.

### 2.8. Rheological Assessment

Rheological measurements were performed using a rotational rheometer (Kinexus Pro+, Netzsch, Selb, Germany) equipped with a 20 mm parallel plate geometry and a 1 mm gap. Samples were tested at 25 °C after a 5 min equilibration period. An amplitude sweep was performed to establish the linear viscoelastic region, followed by a frequency sweep from 0.1 to 10 Hz. Storage modulus, loss modulus, complex viscosity, and tan δ were recorded at 1 Hz and 1% strain.

The undiluted materials evaluated were the test CaHA formulation, non-cross-linked hyaluronic acid (Synovia, CGBIO Co., Ltd., Seongnam, Republic of Korea), and semi-cross-linked hyaluronic acid (Midfoint, CGBIO Co., Ltd., Seongnam, Republic of Korea). Saline was used as the aqueous diluent.

Facetem was mixed with saline, non-cross-linked hyaluronic acid, or semi-cross-linked hyaluronic acid at CaHA to diluent ratios of 1:0.1, 1:0.2, 1:0.3, 1:0.4, 1:0.5, 1:1, 1:2, and 1:3 by volume. Each mixture was homogenized through a Luer lock connector using 10 passes before measurement.

### 2.9. Statistical Analysis

Continuous variables were summarized as mean ± standard deviation across three independent production lots. Measurements obtained from the three syringes within each lot were averaged to generate a single lot value, and lot was treated as the independent experimental unit. Individual particle measurements within each lot were considered nested technical observations and were not treated as independent replicates. Comparisons between the two CaHA formulations were performed using independent-samples t tests based on lot means. Effect estimates were calculated with 95% confidence intervals and two-sided P values. Comparisons involving the PLLA, PLA, and PCL reference materials were descriptive. No formal correlation between viscosity or density and sedimentation was performed because paired measurements across both CaHA formulations were unavailable. Statistical analyses were performed using IBM SPSS Statistics version 29.0 (IBM Corp., Armonk, NY, USA).

## 3. Results

### 3.1. Composition and Microsphere Morphology

Facetem contained 30% CaHA microspheres suspended in a 70% CMC gel carrier. Field-emission scanning electron microscopy showed predominantly rounded microspheres with well-preserved spherical contours. At ×2000, the microsphere surfaces displayed an overlapping granular architecture rather than a completely smooth and featureless shell. Radiesse showed greater surface irregularity and less consistent microsphere contours after removal of the carrier material (Figure 1). The ×5000 image used to characterize Facetem’s lattice-pore surface architecture is presented separately in Figure 2A.

### 3.2. Comparative Particle Characteristics

As shown in Table 1, the mean particle diameters were 34.60 μm for Facetem and 34.90 μm for Radiesse. Their span values were 0.86 and 0.87, respectively, indicating similarly narrow distributions centered predominantly within the 25–45 μm range. Specific surface area was 177.00 m^2^/kg for Facetem and 174.30 m^2^/kg for Radiesse. The corresponding uniformity indices were 0.51 and 0.58. Mean circularity was 0.95 ± 0.03 for Facetem and 0.88 ± 0.07 for Radiesse. Mean roundness was 0.96 ± 0.02 and 0.85 ± 0.08, while mean aspect ratio was 1.04 ± 0.02 and 1.15 ± 0.09, respectively. Particles with a major-axis diameter below 20 μm accounted for 2.10% of Facetem and 14.50% of Radiesse.

In the secondary material comparison, PLLA, PLA, and PCL reference formulations had mean particle sizes of 51.40, 24.90, and 38.40 μm, respectively. Their span values were 1.62, 1.17, and 0.61. Specific surface area was 151.80 m^2^/kg for PLLA, 701.40 m^2^/kg for PLA, and 140.20 m^2^/kg for PCL. The corresponding uniformity indices were 0.56, 0.37, and 0.19 (Table 2).

### 3.3. Comparative Lattice-Pore Morphology and Stability in PBS

At ×5000, the test microspheres showed closely arranged micrograin domains that formed an overlapping lattice-pore surface structure. After 12 weeks of PBS incubation, the fine surface pattern was less distinct and the outer micrograin layer was more irregular, while the overall spherical contour remained identifiable (Figure 2). These findings describe surface morphological change under PBS conditions and do not demonstrate enzymatic, cell-mediated, or in vivo degradation.

### 3.4. Mineral Composition and Gross Homogeneity

X ray diffraction identified Hydroxylapatite as the predominant crystalline phase, accounting for 99.09% of the total mineral content. Inductively coupled plasma optical emission spectroscopy identified calcium and phosphorus as the principal elements. Iron and manganese were below their respective detection limits. The Ca/P molar ratio was 1.67 ± 0.02.

**Figure 3:**
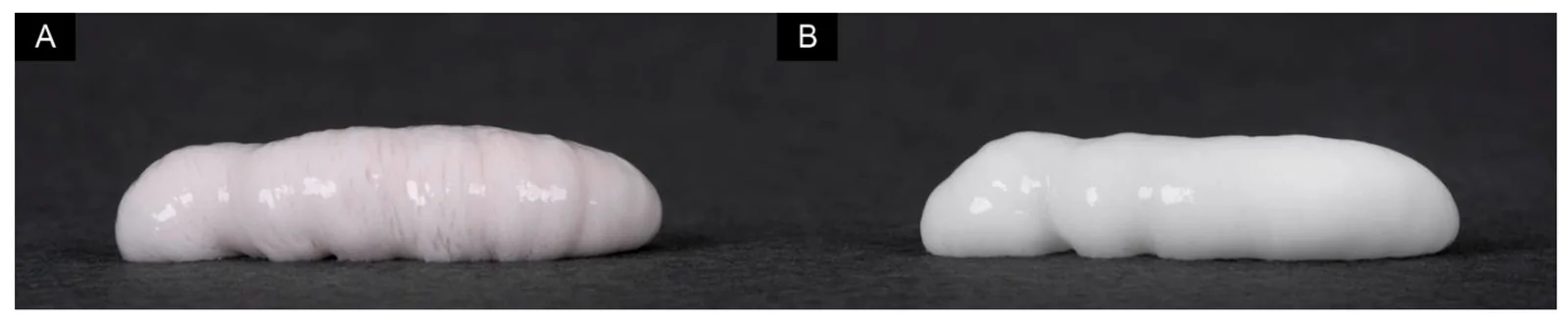
Gross appearance of Radiesse and Facetem after extrusion through a 27G needle. Macroscopic photographs acquired immediately after equal volumes of the formulations were extruded through a 27G needle. (**A**) Radiesse. (**B**) Facetem.

Under standardized imaging conditions, the extruded Facetem appeared uniformly white. Visual color was recorded as a descriptive characteristic of the formulation. After extrusion through a 27G needle, Facetem formed a continuous strand without visible separation between the microsphere phase and the gel carrier. Radiesse showed visible regions of separation between the carrier and particles (Figure 3).

### 3.5. Sedimentation Behavior

**Figure 4:**
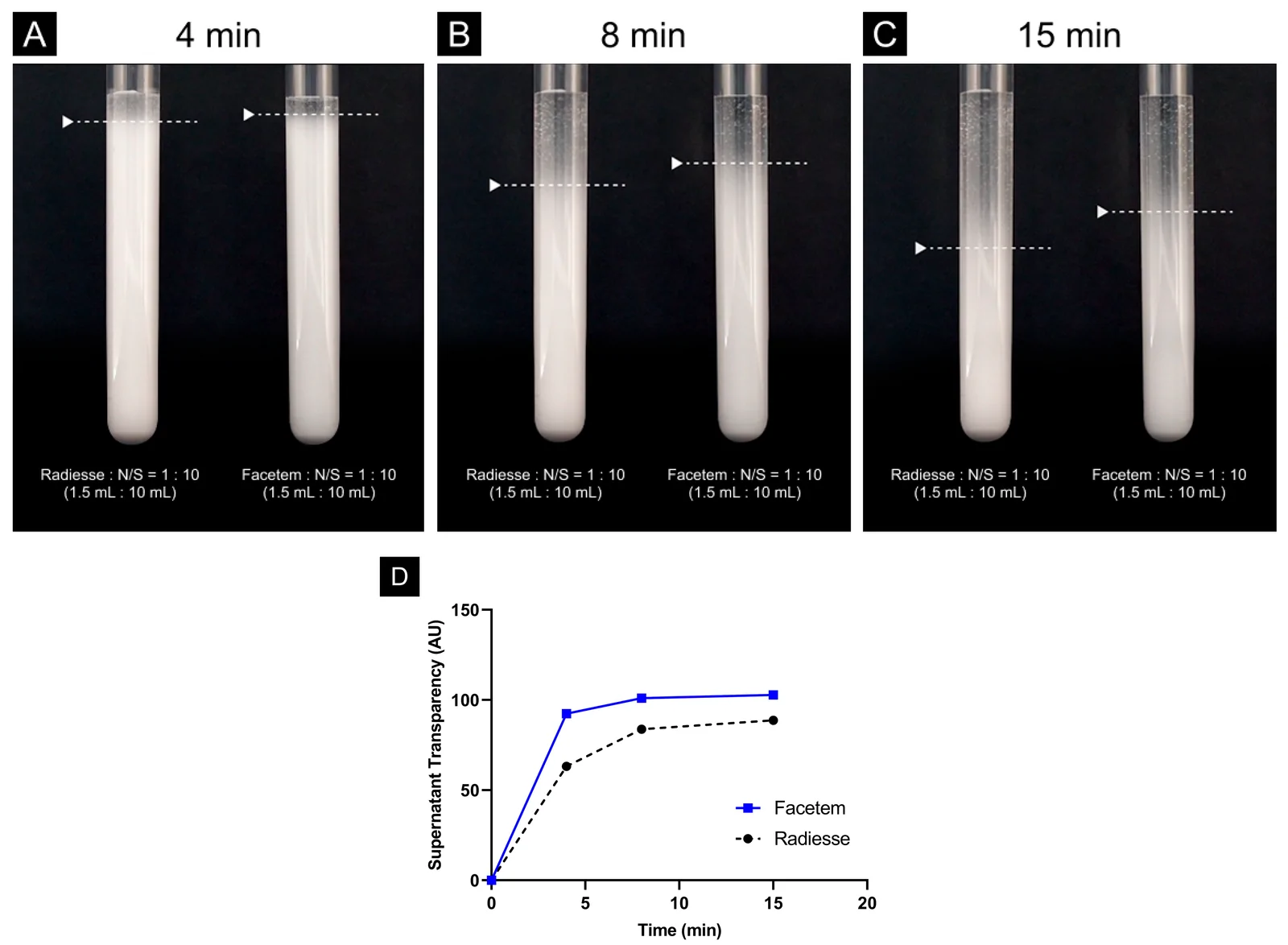
Comparative sedimentation behavior and supernatant transparency of Radiesse and Facetem after saline dilution. Radiesse and Facetem were mixed with 0.9% sodium chloride at a CaHA-to-saline ratio of 1:10. (**A**) Sedimentation at 4 min. (**B**) Sedimentation at 8 min. (**C**) Sedimentation at 15 min. Radiesse is shown on the left and Facetem on the right in each panel. Dashed arrows indicate the boundary between the supernatant and sediment. (**D**) Enlarged plot of supernatant transparency measured by optical densitometry over 15 min.

Both CaHA formulations showed progressive sedimentation after dilution with saline at a ratio of 1:10. Differences in supernatant clarification were visible at 4, 8, and 15 min (Figure 4). These observations represent formulation-level separation behavior under the specified dilution and time conditions.

As shown in Table 3, at 15 min, supernatant transparency was 102.8 AU for Facetem and 88.7 AU for Radiesse, corresponding to an absolute difference of 14.1 AU. Relative to baseline, transparency increased by 46.6% for Facetem and 38.6% for Radiesse, corresponding to a difference of 8.0 percentage points.

When Facetem was normalized to 100%, the relative residual turbidity index of Radiesse was 119.9%. At the 1:2 ratio, Facetem showed a more clearly defined boundary between the supernatant and sediment than Radiesse. The direction of the difference was maintained under the lower-dilution condition, although this condition was evaluated visually. Because paired viscosity or density measurements were not available for both products, these observations were not used to infer a causal relationship between a single material variable and sedimentation or to claim in vivo formulation stability.

### 3.6. Dilutional Rheology

As shown in Table 4, the undiluted Facetem had a storage modulus of 1220.00 Pa, a loss modulus of 627.70 Pa, a complex viscosity of 218.40 Pa·s, and a tan δ of 0.51. Storage modulus exceeded loss modulus, indicating predominantly elastic behavior under the evaluated conditions. Semi-cross-linked hyaluronic acid had a storage modulus of 15.88 Pa, a loss modulus of 12.36 Pa, a complex viscosity of 3.20 Pa·s, and a tan δ of 0.78. The corresponding values for non-cross-linked hyaluronic acid were 0.76 Pa, 1.96 Pa, 0.33 Pa·s, and 2.58.

**Figure 5:**
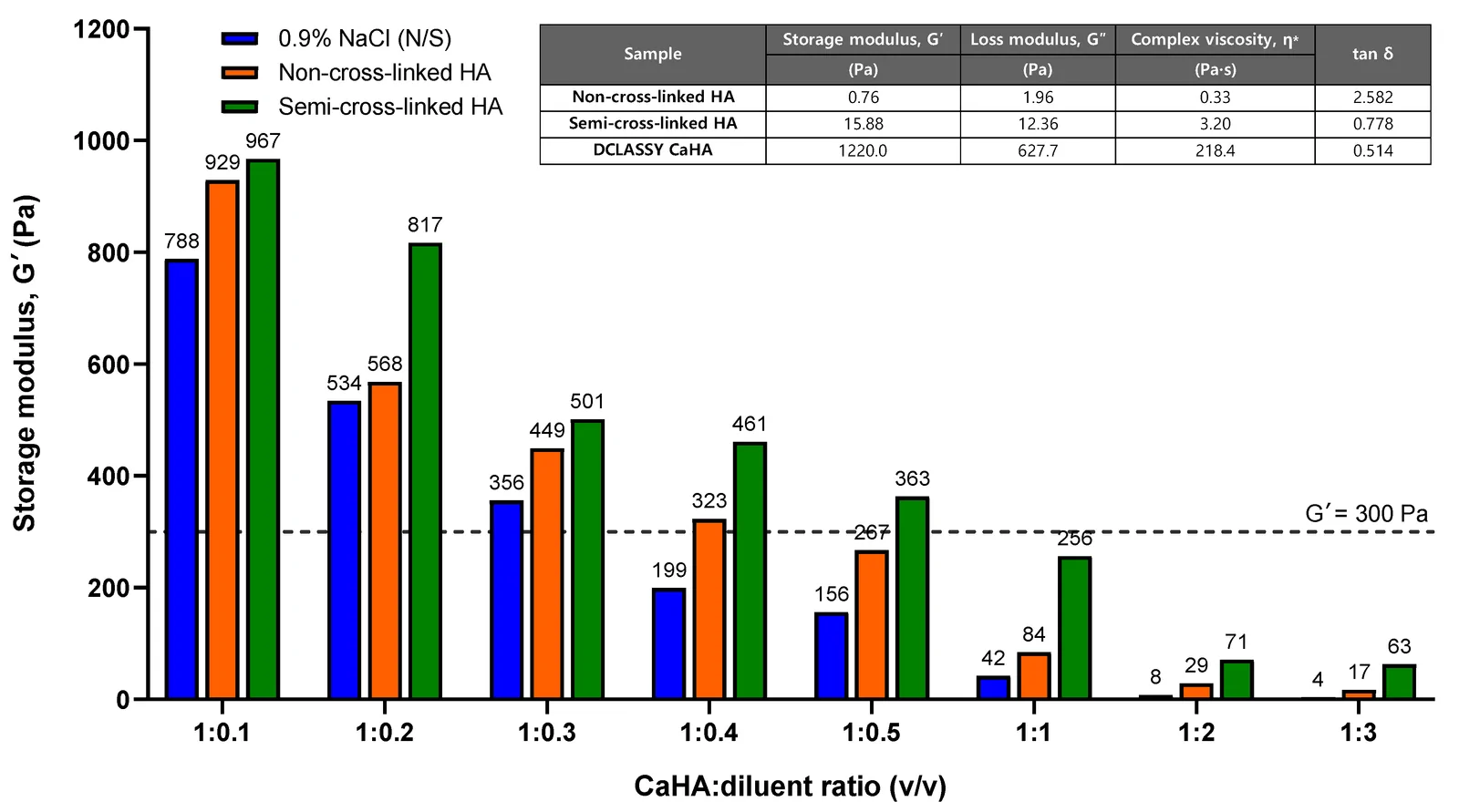
Storage modulus of Facetem according to diluent type and dilution ratio. Storage modulus (G′) of Facetem after mixing with 0.9% sodium chloride, non-cross-linked hyaluronic acid (Synovia), or semi-cross-linked hyaluronic acid (Midfoint) at CaHA-to-diluent ratios ranging from 1:0.1 to 1:3. Bars indicate the measured G′ value at each dilution ratio. The inset table presents the storage modulus, loss modulus, complex viscosity, and tan δ of undiluted Facetem, Synovia, and Midfoint under the evaluated conditions.

Storage modulus decreased progressively as the dilution ratio increased, and the magnitude of reduction varied according to the diluent (Table 5). At 1:0.2, storage modulus was 533.84 Pa with saline, 568.33 Pa with non-cross-linked hyaluronic acid, and 817.36 Pa with semi-cross-linked hyaluronic acid. These values represented retention of 43.8%, 46.6%, and 67.0% of the undiluted storage modulus. At 1:3, storage modulus decreased to 3.50 Pa with saline, 17.16 Pa with non-cross-linked hyaluronic acid, and 63.05 Pa with semi-cross-linked hyaluronic acid. These values represented retention of 0.29%, 1.41%, and 5.17%, respectively. At the 1:3 ratio, the storage modulus of the semi-cross-linked hyaluronic acid mixture was 18.0 times that of the saline mixture and 3.68 times that of the non-cross-linked hyaluronic acid mixture (Figure 5).

## 4. Discussion

This study addressed whether a CaHA formulation prepared using Lattice Pore Formation technology exhibits an internally consistent physicochemical profile across particle geometry, surface architecture, mineral composition, suspension behavior, and dilutional rheology. Its principal contribution is not a single isolated metric but the integrated assessment of these domains within one formulation, with selected morphology and sedimentation comparisons against a reference CaHA-CMC product [8,9,15].

Although the two CaHA formulations had similar mean particle diameters, the f showed a more geometrically consistent microsphere population, with higher circularity and roundness, a lower aspect ratio, and a smaller proportion of particles below 20 μm. The secondary PLLA, PLA, and PCL data further showed that mean diameter, distribution span, specific surface area, and uniformity are distinct material descriptors. The distinguishing feature of Facetem was therefore the combination of regular geometry and a limited small-particle fraction rather than mean particle size alone.

Particles at or below approximately 20 μm may be more readily taken up by phagocytic cells, and local curvature influences particle-cell engagement [16,17]. The smaller fraction of particles below 20 μm and the more uniform geometry therefore define a more homogeneous particulate population. These in vitro material findings do not by themselves establish a clinical or cellular advantage; rather, they provide a physicochemical basis for future biological testing. Direct contact between CaHA microspheres and fibroblasts and new collagen formation around dispersed microspheres have been reported in prior studies [4,18,19].

The lattice-pore surface showed morphological alteration after 12 weeks of PBS incubation while the principal spherical contour remained recognizable. Because PBS lacks enzymes and cells, this assay cannot establish a degradation mechanism, biodegradation kinetics, in vivo persistence, or biological remodeling. It is therefore interpreted as a morphological-stability test under non-biological buffer conditions. Radiesse was not included in this 12-week assay, so the findings are within-product observations and not evidence of comparative degradation performance.

The organized surface was present within a chemically well-defined mineral phase. Hydroxylapatite accounted for 99.09% of the mineral content, and the Ca/P molar ratio of 1.67 matched the stoichiometric ratio reported for hydroxylapatite and an independently synthesized CaHA-CMC matrix [20]. Surface architecture and mineral composition are separate properties because formulations based on the same crystalline phase may differ in contour and surface organization [6,7,21]. The present results show that the observed lattice-pore surface architecture was obtained without departure from the mineral composition expected of CaHA.

At the formulation level, Facetem extruded as a continuous strand and showed a clearer supernatant and sharper sediment boundary under the standardized dilution assay. Sedimentation depends jointly on particle shape, particle and carrier density, surface properties, particle concentration, and carrier viscosity. Prior work on injectable calcium-phosphate suspensions similarly showed that settling kinetics and sediment compactness vary with particle size and polymer concentration or viscosity [22]. Because paired viscosity or density measurements were not available for both CaHA products, the present study cannot identify which variable drove the observed difference, and no correlation was calculated. The sedimentation findings should therefore be interpreted as descriptive formulation-level separation behavior under the tested conditions, not as proof of baseline formulation homogeneity, in vivo stability, tissue distribution, or clinical superiority.

Rheological analysis showed that storage modulus and complex viscosity decreased with increasing dilution, consistent with prior studies of CaHA-CMC formulations [12,13,15]. At equivalent dilution ratios, semi-cross-linked hyaluronic acid retained more elastic resistance than non-cross-linked hyaluronic acid, which in turn retained more than saline. These results show that diluent identity, not only dilution volume, changes the mechanical profile of the final mixture. Because the rheological analysis was performed for Facetem and its diluent mixtures, it should not be interpreted as a head-to-head rheological comparison with Radiesse.

From a material-characterization perspective, the undiluted state provided the greatest resistance to deformation, whereas increasing dilution produced progressively lower modulus values. Clinical consensus and the rheological literature have discussed such mechanical profiles in relation to different injection strategies [10,11,21,23,24,25]. The present study did not assess clinical outcomes, injection force, anatomical-layer placement, tissue distribution, or safety and therefore does not establish suitability for any specific indication or treatment objective. The in vitro results should not be interpreted as comparative clinical efficacy.

Taken together, the findings define a formulation-specific material profile in which particle geometry, lattice-pore surface architecture, mineral composition, sedimentation behavior, and diluent-dependent rheology were evaluated within the same analytical framework. The more consistent microsphere geometry, distinct lattice-pore surface architecture, and continuous strand extrusion distinguish Facetem at the material level and support its description as a next-generation CaHA formulation. This integrated approach is the central novelty of the work. The revisions deliberately distinguish direct observations from mechanistic or clinical inference and limit conclusions to the conditions actually tested.

### Study Limitations

This study has several limitations. The 12-week PBS assay lacked enzymes and cells and included only Facetem. The sedimentation comparison was not accompanied by paired viscosity or density measurements for both CaHA products, precluding correlation or causal attribution. Rheological analysis characterized Facetem and diluent mixtures rather than both CaHA formulations head-to-head. Extrusion homogeneity and the 1:2 sedimentation condition were assessed qualitatively. Finally, this in vitro study did not evaluate biological response, clinical efficacy, injection force, tissue distribution, or safety. Conclusions were revised accordingly.

## 5. Conclusions

Within the tested conditions, Facetem showed a relatively uniform microsphere population, an organized lattice-pore surface, hydroxylapatite mineral composition with a Ca/P ratio of 1.67, continuous extrusion, distinct sedimentation behavior, and diluent-dependent rheology. This physicochemical profile supports the characterization of Facetem as a next-generation CaHA formulation defined by controlled microsphere geometry and a distinct lattice-pore surface architecture. The integrated analytical framework is the study’s main contribution. The PBS findings indicate morphological change during non-biological incubation rather than in vivo degradation, and the comparative sedimentation data remain descriptive because paired viscosity or density measurements were not available.

## Figures and Tables

**Table 1:** Quantitative particle characteristics of the two CaHA formulations.

Parameter	Facetem	Radiesse
Mean particle size, μm	34.60	34.90
Span	0.86	0.87
Specific surface area, m^2^/kg	177.00	174.30
Uniformity index	0.51	0.58
Circularity	0.95 ± 0.03	0.88 ± 0.07
Roundness	0.96 ± 0.02	0.85 ± 0.08
Aspect ratio	1.04 ± 0.02	1.15 ± 0.09
Particles below 20 μm	2.10%	14.50%

**Table 2:** Particle characteristics of CaHA and polymer reference materials.

Material	Composition	Mean Particle Size, μm	Span	Specific Surface Area, m^2^/kg	Uniformity Index
Facetem	CaHA	34.60	0.86	177.00	0.51
Radiesse	CaHA	34.90	0.87	174.30	0.58
PLLA reference	PLLA	51.40	1.62	151.80	0.56
PLA reference	PLA	24.90	1.17	701.40	0.37
PCL reference	PCL	38.40	0.61	140.20	0.19

**Table 3:** Sedimentation and supernatant transparency at 15 min.

Parameter	Facetem	Radiesse
Supernatant transparency, AU	102.8	88.7
Increase from baseline	46.6%	38.6%
Relative residual turbidity index	100.0%	119.9%

**Table 4:** Rheological properties of the undiluted materials.

Material	G′, Pa	G″, Pa	Complex Viscosity, Pa·s	tan δ
Facetem	1220.00	627.70	218.40	0.51
Semi-cross-linked HA	15.88	12.36	3.20	0.78
Non-cross-linked HA	0.76	1.96	0.33	2.58

**Table 5:** Storage modulus according to diluent and dilution ratio.

CaHA: Diluent Ratio	0.9% NaCl, Pa	Non-Cross-Linked HA, Pa	Semi-Cross-Linked HA, Pa
1:0.1	788.41	929.01	967.16
1:0.2	533.84	568.33	817.36
1:0.3	356.00	448.83	501.47
1:0.4	199.45	322.80	461.14
1:0.5	156.32	266.85	363.30
1:1	42.43	83.96	256.26
1:2	8.03	28.59	71.25
1:3	3.50	17.16	63.05

## Data Availability

The data generated and analyzed during this study are available from the corresponding author upon reasonable request.
